# Proposed Test for the Detection of Impure Chloroform

**Published:** 1849-07

**Authors:** T. Cattell


### PROPOSED TEST FOR THE DETECTION OF IMPURE CHLOROFORM.

BY T. CATTELL.

The extract from the *Pharmaceutical Journal*, in the Lancet of the 8th ult., headed "Properties and Test of the purity of Chloroform," cannot, I think, be regarded as conclusive or strictly scientific.

1. It is stated that impure chloroform possesses vesicating properties when applied to the skin or mucous membrane, whereas the pure liquid produces simple redness only—this is not correct. I have chloroform in which the presence of alcohol can be detected, but which will not induce any irritating action when applied to the skin. That chloroform may be made to produce a vesicating effect; I admit, but this is perfectly independent of any notion respecting its purity; the effect is referrible solely to the prevention of its evaporation, and of its limitation to a particular part. Did chloroform possess this property in a vaporized state, then, like the vapor of liquor of ammonia, it would, if inhaled, excite the capillaries of the mucous membrane into abnormal action; but this is not the case—its causticity has therefore no connexion either with its purity, or with its use as an anæsthetic agent.

2. The same remark applies to the opalescence said to be occasioned by dropping chloroform into distilled water. A short time since, when in town, I called upon a highly respectable manufacturer of chloroform, and observing two bottles of the liquid, one being opalescent, and the other not, I inquired into the cause of the difference, and was informed that it was owing to the chloroform having been put into the bottle not perfectly dry; but had it been first rinsed with a little spirits of wine, the fluid would have remained quite clear.

Suspecting from this that the chloroform which had been purchased contained alchohol, I tried the following experiments:

(*a*) To about two drachms of chloroform were added a cyrstal or two of chromic acid, which became, after a few moments' agitation, changed into the green oxide of chrome, a result positively indicative of the presence of alcohol.

(*b*) To the same quantity of chloroform was added a small quantity of bichromate of potash and sulphuric acid; the green oxide of chrome being produced in this as in the other instance.

The experiments were repeated, and with precisely analogous results.

P. S.—Enclosed is a slip of paper, stained with the green oxide of chrome, obtained from about twenty drops of chloroform. If held opposite to an artificial light after dark, the green stain will be as evident as by natural light.

